# Obstetrics, and Diseases of Women and Children

**Published:** 1860-01

**Authors:** Wm. B. Atkinson

**Affiliations:** Lecturer on Obstetrics and Diseases of Women and Children


### OBSTETRICS, AND DISEASES OF WOMEN AND CHILDREN.

BY WM. B. ATKINSON, M.D.,
LECTURER ON OBSTETRICS AND DISEASES OF WOMEN AND CHILDREN.

### I.—*The Sickness of Pregnancy.* By Dr. C. E. Bagot.

1. Dr. Bagot relates a case in which, after every other remedy had been unsuccessfully employed, he had recourse, with excellent results, to the constitutional effects of mercury. As soon as salivation supervened, the vomiting ceased, food was retained, the patient rapidly recovered, and the gestation went on in a natural manner. The same patient, on two subsequent occasions, was similarly attacked, and each time the employment of calomel produced the happiest results. On the last occasion the illness was of the worst form, and fears were entertained for her safety. Dr. Bagot expresses the hope that a fair trial will in future be given to this remedy, prior to the induction of premature labor.—(*Dublin Med. Press*, October 12, 1859.)

2. Dr. Warren, of Neponset, has, in this complication of pregnancy, employed, with highly satisfactory results, a combination of Tinct. of Benzoin and Chloric Ether, as an application to the mouth of the womb. This he finds especially beneficial when there is neuralgia and excessive leucorrhœal secretion. His formula is:—

℞.—Tinc. Benzonii, c., f℥ij;

Chloric Ether, f℥i;

Acet. Morph., grs. ij.

This is applied, by means of a brush, once in three or four days.

He also speaks favorably of the nitrate of silver, applied lightly to the *os uteri*, and injections of:—

℞.—Ferri Aluminis, f℥i;

Inf. Opii, f℥ij;

Aquæ, f℥viij.

For the alum, the iodide of zinc may be substituted in cases where a spasmodic action is observed in the womb or the neck of the bladder. This may be added in the proportion of five grains to the ounce.

He regards the good effects of these applications as arising from "the protecting coat which they form over the congested and abraded surface."—(*Boston Med. and Surg. Journal*, September 1, 1859.)

### II.—*Uva Ursi as a Parturifaciant.* By Dr. Strube, of New Orleans.

As, by some obstetricians ergot is thought to act too powerfully on certain occasions, and as it sometimes fails in producing the desired effects, either from worthlessness, or from some idiosyncrasy of the patient, it has been somewhat of a desideratum to obtain a substitute for this remedy, which has hitherto appeared to stand almost alone.

Dr. Gauchet, of Paris, highly recommends for this purpose the leaves of the *Uva Ursi*, in the form of a strong decoction, of which a teacupful is to be administered every half hour. He relates a number of cases in which this agent acted promptly, producing an increase of pains without any of that sudden acceleration which too frequently occurs after the use of secale cornutum. We would suggest a trial of this article, and a report of the results obtained.—(*N. O. Med. and Surg. Journal*, November, 1859.)

### III.—*Records of Midwifery Practice.*

1. *An Analysis of Two Thousand Consecutive Cases in Midwifery.* By G. RIGDEN, Esq., etc. This paper was read before the East Kent and Canterbury Medical Society, and gives the following valuable facts: In the 2000 labors, 2025 children were born. One mother gave birth to twins at four consecutive confinements. Four mothers died from the complications or consequences of labor; one from convulsions and coma two hours after delivery, and three from puerperal fever, occurring five, six, and fourteen days respectively after labor. Ninety-six children were still-born, forty-six males and fifty females. Four cases had retained placenta, three from inertia and one from irregular contraction of the uterus. Five, after labor, had retention of urine, but all recovered. Five mothers had puerperal convulsions; one died. Twenty-six of the labors were with breech presentation; all delivered naturally; eight of the children died. Fourteen were face presentations; five of the children died; eleven of these cases were delivered naturally. Twelve cases were shoulder or arm presentations, and all were delivered by turning; five of the children being still-born. There were six cases of placenta prævia; all delivered by turning; all the children premature and still-born. Nine were cranial presentations, delivered by the forceps; six children being saved. Five mothers subsequently had puerperal fever; three died. One had puerperal mania, terminating in recovery after six months. Eight children were more or less deformed. Prolapsed funis, two cases. Of the twins, there were: both males, six; both females, twelve; male and female, seven.—(*Brit. Med. Journal*, October 29, 1859.)

2. *One Thousand Cases of Obstetrics.* By J. S. HARRINSON, Esq. This paper was read before the Reading Pathological Society, and gave the following data:—

| | |
|---|---|
| In mothers between 15 and 19 years, . . . . . | 11 |
| " " 20 " 24 " . . . . . . | 176 |
| " " 25 " 29 " . . . . . . | 263 |
| " " 30 " 34 " . . . . . . | 269 |
| " " 35 " 39 " . . . . . . | 192 |
| " " 40 " 44 " . . . . . . | 80 |
| " " 45 " 49 " . . . . . . | 9 |

November, December, and January had more than any other three consecutive months. May had the largest; April, the smallest. Classifying them according to the hours, there were 270 occurring between 12 P.M. and 6 A.M.; 268, from 6 A.M. to 12 M.; 214, from 12 M. to 6 P.M.; and 248, from 6 P.M. to 12 P.M.

The duration of pregnancy averaged about 280 days. The average duration of labor was 7·36 hours; first labors being, as is usual, much longer. The 1000 labors resulted in the birth of 1010 children; 504 males, 506 females. Breech and feet presentations numbered 39; face, 17; with an extremity, 121. Lingering labors, 15; instrumental, 17; preternatural and complicated, 61.—(*Ibid.*)

3. *Thirteen Hundred Cases of Midwifery.* By A. Smith, Esq., etc. In these 1300 cases, 1320 children were born; 700 males, 620 females; still-born, 65; premature births, 38. Presentations of the breech, 23; of the face, 2; forehead, 20; extremities, 9. Prolapse of the funis occurred in 6 cases. Placenta prævia, complete, 3—children all still-born; partial, 2—one child dead. Three mothers had convulsions; three had peritonitis; one, mania. Version was performed in 4; the forceps were used in 25; craniotomy in 3. There were no cases of retention of the placenta, or of severe post partum hemorrhage. He attributes the absence of these complications to the practice of applying a binder as soon as the second stage of labor commences, tightening it after delivery of the child, and again after the expulsion of the placenta, and abstaining from the use of ergot. The number of forceps cases is great, but no bad results have followed, and he believes their timely employment prevents laceration of the perinæum, rather than causes it.—(*Lancet*, November 12, 1859.)

4. *Still-born Children.* By J. Hardaway, Esq., etc. This paper gives 732 cases, in which 72 children were still-born, showing, in the aggregate, about ten per cent. No other statistics are given.

5. *Statistics of Four Thousand and Forty-nine Cases of Midwifery.* By R. Dunn, F.R.C.S., etc. Of these, there were 228 premature births; 2133 children were males, 1688 females; 2 cases of triplets, and 45 of twins; 3 cases of monstrosity. There were 170 still-born; 60 cases of preternatural presentation. Prolapse of the funis, 11; of which 8 were born dead. Breech presentations, 25; still-born, 9. Face presentations, 3; face to pubis, 11. He had 10 cases of craniotomy; 6 cases of placenta prævia; 30 of adherent placenta; 12 cases of puerperal fever, 3 died; 4 cases of puerperal convulsions, all recovered. Phlegmasia dolens occurred in 6 cases, and 2 proved fatal; scarlatina in 3, and 1 died.

IV.—*The Treatment of Menorrhagia, Leucorrhœa, etc., by Arsenic.* By A. P. Burns, M.D., Ellicott's Mills, Md.

In cases of menorrhagia, leucorrhœa, hemorrhage in threatened abortion, and after delivery, and excessive lochial discharges, arsenic has proved highly serviceable. In the hands of Dr. Burns, it has never failed, he having long been in the habit of using it for these affections, and particularly in the form of Fowler's solution. The dose is ten drops every fifteen or twenty minutes, till hemorrhage is checked; after which he administers it in five-drop doses three times a day. He has even found it to suspend the menstrual secretion. In leucorrhœa, he gives five drops three times a day, persevering until a cure is effected.

Occasionally he has recourse to injections, or counter-irritation by blisters to the sacrum. When the patient is much debilitated, he adds to this treatment the following:—

℞.—Tr. Cinchonæ, c., f℥iij;

Tr. Cantharidis, fℨij.

Dose, a teaspoonful three times a day. He relates some cases to show the results of his treatment.

This remedy has been *continued without intermission for months, without any unpleasant effects.*

He says: "I know of no remedy so effective and so prompt in arresting hemorrhage in threatened abortion; it seems to suspend at once the contractions as well as the hemorrhage." Hence it cannot act by producing contractions of the uterus. It is a hæmostatic of great power, having succeeded equally well in hæmoptysis, etc.

In profuse lochial discharges, when this remedy is combined with the tonic mixture above, it acts promptly, even where every other remedy has failed, and the case has gone on for some weeks without amendment.—(*Am. Jour. Med. Sci.* October, 1859.)

V.—*Chronic Inversion of the Uterus.* By DR. F. RAMSBOTHAM, of London.

The following case is related by Dr. Ramsbotham, of the London Obstetric Hospital. It is interesting as showing the possibility of spontaneous replacement of an inverted uterus, after it had been displaced for some months, and even after some very rough treatment:—

"I was sent for on July 20, 1839, to see a young lady of relaxed fibre and cachectic habit, who had been delivered of her first child, after a severe labor, twelve hours before, and whom I found suffering from violent forcing-pain and great hemorrhage. She was exceedingly depressed. I detected a tumor, occupying the vagina, as large as a man's closed fist, entirely covered by a layer of coagulum. It was sensitive, though not painfully so, possessed a doughy feel, and became harder when compressed. The uterus could not be felt in the abdomen; but above the pubes there was a sensation of a most unusual void. As she had passed no urine since delivery, I relieved the bladder; and having no doubt that the tumor was the uterus inverted, I made strenuous efforts to restore it, without effect, but not without putting her to considerable pain. After some time I desisted in despair, fearing that I should lacerate the upper part of the vagina; for the tumor had become hard while I was making these efforts, and, in the same proportion, the circle of the mouth became closed around it in the form of a forcibly constricted ring. She passed a quiet night under the influence of morphia, and in the morning voided urine naturally, with some coagula. She was confined to bed for two, and to the house for three months, with severe lumbar pains, and copious irregular hemorrhages. From the middle of October till the end of December, she was free from flooding, but still annoyed by bearing-down pains, attended by a profuse, glairy, leucorrhœal discharge. The hemorrhage returned, and continued for two months, after which she was moved into the country, and I did not see her again till May twenty-second. She was then in such imminent danger that, in consultation with Mr. Hamilton, at that time assistant surgeon to the London Hospital, and with her general attendant, it was agreed to remove the tumor by ligature as soon as she was a little recruited. It was at this time the size of an ordinary nonpariel apple, and had every characteristic of an inverted uterus. On June fifth, with the assistance of the gentlemen mentioned above, I placed a ligature round its upper part by means of the double canula. The application gave but little pain at the moment, and the bleeding, which had been going on almost uninterruptedly, ceased immediately. In three or four hours, however, a violent rigor supervened; this was followed by symptoms of intense peritoneal inflammation, and the ligature was removed twenty hours after its application. The pain and other inflammatory symptoms gradually subsided; in a few days she was able to leave her room; she menstruated on July thirteenth less profusely than she had been accustomed to do, and continued regular in that respect; she regained her flesh, color, and appetite; was able to take a long walk, had no bearing down, nor difficulty in passing water; and, when I saw her in January, 1841, she told me that she enjoyed better health than she had done for many years. Nothing solid had ever passed from the vagina since the operation. Early in the year, symptoms of pregnancy manifested themselves; and I delivered her of a six months' fœtus on July seventh, in the same year, 1841. Since then I have also attended her with four other children. On one occasion the placenta adhered, and I had to introduce my hand to remove it. I sought for a polypus in the cavity, but there was nothing like one. On another occasion, after the placenta had been expelled naturally, she was harassed with violent spasmodic bearing-down pains, which induced me to pass my hand into the uterus; I there found the fundus and posterior part of the body protruded considerably downward and forward, there existing evidently a disposition for inversion to occur again; this, however, was obviated by the introduction of the hand." This ought to give us additional confidence in any effort we may undertake for the purpose of remedying, by manipulation, this serious accident.—(*Med. Times and Gazette*, November 5, 1859.)

VI.—*Treatment of Pelvi-Peritonitis.* By DR. TILT.

In concluding an article on the "Relation of Peritonitis to Uterine Pathology," Dr. Tilt says: "I am afraid that our larger knowledge of the diseases peculiar to women has not in every instance led to their better treatment. However wrong were our predecessors in giving the name of 'inflammation of the bowels' to so many different diseases, still the name led to the enforcing of perfect repose, of low diet, warm poultices, and leeches—treatment which I still believe is the most appropriate, notwithstanding the disrepute into which venesection has fallen, and the spreading of the dangerous doctrine that a stimulant system constitutes the best means of treating fevers and inflammations."

In pelvi-peritonitis, he applies leeches and gives calomel and opium according to the strength of his patient. Ointments of mercury and belladonna may be freely rubbed over the abdominal walls, and warm poultices, frequently renewed. Mild purgation will also be found eminently useful. To the anæmic he gives steel and quinia.—(*Lancet*, September 10, 1859.)

VII.—*Hydatidiform, or Vesicular Mole; its Nature and Mode of Origin.* By GRAILY HEWITT, M.D., etc.

The author of this paper endeavors to reduce the recorded facts in reference to the vesicular mole to a system, as well as to solve questions not hitherto satisfactorily answered. He describes a case, where the mole was expelled seven months subsequently to the birth of a first child, and during lactation. The ovum was about two months old, and presented no appearance of an embryo.

After some remarks in reference to the solutions offered concerning these bodies, the author gives his views. He considers that the death of the embryo occurs, which is followed by the transformation which is found in the chorion. Death arrests the development of the chorion, but not of the chorion villi. These continuing to grow from the hydatidiform mole, the third month being probably the limit within which this change can commence. Generally, no traces of the embryo are present; when otherwise, its size is extremely small. In particular cases he believes the death of the embryo to be due to a long protracted contraction of the uterus, arising from some irritation, as that of lactation. He believes that the placenta of a mature fœtus cannot be changed into a mole. Where such appears to have occurred, it is produced by a double conception, and one ovum having perished early; or, perhaps, a portion of the chorion villi being accidentally detached from the embryo, becomes thus changed. He was inclined to admit the possibility of the expulsion from the uterus of a true hydatid. The fact that in true hydatids we find cysts inclosed one within another, and in the other case round or oval bodies attached one to another, like beads, would be alone sufficient to prevent the possibility of a mistake in the diagnosis.

DR. BARNES, in these cases, had observed a tendency to fatty degeneration. The absence of the embryo was accounted for, by its undergoing oily transformation and dissolution. He had witnessed this in progress. He thought it not established that the death of the fœtus necessarily preceded the change, as fatty degeneration sometimes preceded the fœtal death.

DR. DRUITT agreed with the author in regard to the proposition that cystic disease of the chorion was an exaggeration and deformity of natural structure, though he doubted that death of the fœtus was the only and essential cause. He believed that a sort of apoplectic engorgement of the vessels of the decidua usually preceded abortion; and that the changes of structure were generally secondary, though some were primary. It is an anatomical error to speak of the disappearance of any of the villi of the chorion, whether of the placental or non-placental portion of that membrane. On the contrary, the villi of the whole chorion continue to grow up to the end of pregnancy, and are readily formed in every mature ovum. They are particularly large around the placental portion, and in this part in the membranes at full term he had occasionally found them excessively ampullated, as if in an incipient state of cystic degeneration.—(*London Obstetrical Society, Brit. Med. Journal,* October 22, 1859.)

VIII.—*A Weeping Leg.* By DR. R. S. ORR, of the Royal Glasgow Infirmary.

A married woman of middle age, who had had several children, presented herself with the following singular complaint, viz., a constant discharge of serum from the inside of one of her thighs, her clothes being saturated with it, and increasing on exertion. This had existed for seventeen years, no physician having been previously consulted. It could not be traced to any vicarious discharge. On the thigh, there were a number of well defined vesicles, some entire, some broken. Many had at the apex a minute aperture, from which exuded *guttatim*, a serous fluid of a slightly milky aspect. The fluid was not examined chemically. There was no excoriation or irritation of the skin from this discharge.—(*Glasgow Med. Journal*, October, 1859.)

IX.—*New Mode of Resuscitating Still-born Children*. By Dr. Sylvester.

The "Ready-Method" of Marshall Hall has been very extensively employed both in this country and in Europe, and almost every number of the London *Lancet* contains the record of cases, in which it has been the means of saving life. Nor can it be too strongly insisted upon, where there exists the slightest hope of success. Dr. Sylvester, of London, relates a case of still-birth, where "sprinkling with cold water, etc., produced no effect, probably owing to the insensibility arising from compression of the head by the instrument (the forceps) or from the unusual severity of the labor. The case was given up as hopeless by the by-standers, when I determined to try my method of resuscitation; and, raising the child's arms up by the sides of his head, I extended them gently and steadily upward and forward for a few moments, thus enlarging the capacity of the chest by elevating the ribs through the pectoral muscles. By this means, I induced a tendency to a vacuum in that cavity, and an inspiration of air was the result. Next, by turning down the little patient's arms, and pressing them gently against the sides of the chest, I produced a forced expiration. In less than ten minutes the natural respiration was established, and I am happy to be able to add that the mother and child are progressing favorably."—(*London Lancet*, October, 1859.)

X.—*On the Suction Method of Rearing Infants*. By F. J. Brown, of Chatham.

The object of this paper is to point out the value of milk as food for infants, and the importance of its administration by the suck-bottle, and also to oppose the practice of pap-feeding. Suction is the natural mode by which the child obtains its food, and any other way is extremely liable to produce injurious effects. Spoon-food causes flatulence, eructations, vomiting, and diarrhœa. The acts of suckling should not be repeated oftener than every three hours. Children thrive well on cow's milk diluted with one-sixth water, and slightly sweetened. This should be given warm, and with the child lying in a semi-recumbent position. At each period of suckling, the child should be permitted to take its fill, (four to eight ounces,) thus getting about one or two quarts daily. The child should be early taught to do without feeding for four or five hours in the night.

Prior to the appearance of the teeth, no other food should be given. Nature sends the teeth to indicate that mastication should replace suction. About this period it is proper to give a small quantity of pap, which may be made of white bread (free from alum) scalded or boiled slightly, and sweetened with a small quantity of white sugar. Gradually increase the feeding, and finally give beeftea, gravy, and any wholesome food prepared for the usual meals of the family. When the teeth appear late, the feeding must also be adopted late.

In allusion to sleep, he adds that in all cases infants should sleep in a bed separate from their parents, but near at hand. Many children undoubtedly perish, not by being overlaid, but by asphyxia; the child sinking beneath the clothes, and thus being compelled to breathe an atmosphere highly charged with carbonic acid gas.—(*Brit. Med. Journal*, October 29, 1859.)

